# Arguing against the proposed definition changes of PD

**DOI:** 10.1002/mds.26721

**Published:** 2016-08-05

**Authors:** Bradley F. Boeve, Dennis W. Dickson, John E. Duda, Tanis J. Ferman, Douglas R. Galasko, James E. Galvin, Jennifer G. Goldman, John H. Growdon, Howard I. Hurtig, Daniel I. Kaufer, Kejal Kantarci, James B. Leverenz, Carol F. Lippa, Oscar L. Lopez, Ian G. McKeith, Andrew B. Singleton, Angela Taylor, Debby Tsuang, Daniel Weintraub, Cyrus P. Zabetian

**Affiliations:** ^1^ Department of NeurologyMayo Clinic College of MedicineRochesterMinnesotaUSA; ^2^ Departments of Pathology and NeuroscienceMayo ClinicJacksonvilleFloridaUSA; ^3^ Department of NeurologyUniversity of Pennsylvania School of MedicinePhiladelphiaPennsylvaniaUSA; ^4^ Department of Psychiatry and PsychologyMayo Clinic College of MedicineJacksonvilleFloridaUSA; ^5^ Department of NeurosciencesUniversity of CaliforniaSan DiegoCaliforniaUSA; ^6^ Marcus Neuroscience InstituteFlorida Atlantic UniversityBoca RatonFloridaUSA; ^7^ Department of Neurological SciencesRush University Medical CenterChicagoIllinoisUSA; ^8^ Department of NeurologyHarvard Medical School at Massachusetts General HospitalBostonMassachusettsUSA; ^9^ Department of NeurologyUniversity of Pennsylvania School of MedicinePhiladelphiaPennsylvaniaUSA; ^10^ Department of NeurologyUniversity of North Carolina at Chapel HillChapel HillNorth CarolinaUSA; ^11^ Department of RadiologyMayo Clinic College of MedicineRochesterMinnesotaUSA; ^12^ Lou Ruvo Center for Brain HealthCleveland ClinicClevelandOhioUSA; ^13^ Department of NeurologyDrexel University College of MedicinePhiladelphiaPennsylvaniaUSA; ^14^ Department of NeurologyUniversity of PittsburghPittsburghPennsylvaniaUSA; ^15^ Institute of AgeingNewcastle UniversityNewcastle upon TyneUK; ^16^ Laboratory of Neurogenetics, National Institute on AgingNational Institutes of HealthBethesdaMarylandUSA; ^17^ Lewy Body Dementia AssociationLilburnGeorgiaUSA; ^18^ Department of Psychiatry and Behavioral SciencesUniversity of WashingtonSeattleWashingtonUSA; ^19^ Department of PsychiatryUniversity of PennsylvaniaPhiladelphiaPennsylvaniaUSA; ^20^ Department of NeurologyUniversity of Washington School of MedicineSeattleWashingtonUSA

**Keywords:** Dementia with Lewy bodies, dementia, Parkinson's disease, parkinsonism

## Abstract

As members of the Lewy Body Dementia Association Scientific Advisory Council, we aim to address some of the issues raised in the article titled “Time to Redefine PD? Introductory Statement of the MDS Task Force on the Definition of Parkinson's Disease.” In particular, we suggest that the 1‐year rule distinguishing Parkinson's disease dementia from dementia with Lewy bodies is worth maintaining because it serves an important purpose in clinical practice and clinical and basic science research and when helping the lay community understand the complexity of these different clinical phenotypes. Furthermore, we believe that adding an additional diagnostic label, “PD (dementia with Lewy bodies subtype),” will confuse rather than clarify the distinction between dementia with Lewy bodies and PD or PD dementia, and will not improve management or expedite therapeutic development. We present arguments supporting our contentions. © 2016 The Authors. Movement Disorders published by Wiley Periodicals, Inc. on behalf of International Parkinson and Movement Disorder Society.

We read with interest the article by Berg and colleagues^1^ regarding possible changes to the definition of PD as proposed by the International Parkinson and Movement Disorders Society task force commissioned to consider a redefinition of PD. Comments were sought by the authors regarding the content of this article. We aim to address some of the issues raised in this article, particularly those relating to dementia and the distinction between dementia with Lewy bodies (DLB) and Parkinson's disease dementia (PDD).

We agree that there is clinical overlap between DLB and PDD and concur that the 1‐year time period may not be the optimal method for diagnostic distinction, but we also believe that the rule serves a useful purpose for clinical practice and research studies and that its dismissal would be both premature and problematic. We argue that insufficient new evidence has been presented to justify a modification to the diagnostic criteria for PDD and DLB at this time. We also believe that adding an additional diagnostic label, “PD (DLB subtype),” will confuse rather than clarify the situation and will not improve management or expedite therapeutic development.

## Addressing the “Challenging the Status Quo” Comments

Relevant challenges to redefine PD from the manuscript are restated in italics below, with our responses stated beneath each challenge.

*Similar neuropsychological findings, with predominant visuoperceptual impairment, improvement of memory with cueing, and so on*.

We concur that once the dementia is diagnosed, visual perceptual impairment, attention difficulty, and slowed reaction time occur in both DLB and PDD.^2^, ^3^ However, the comorbid presence of Alzheimer's disease (AD) pathology is not uncommon in DLB and has been shown to contribute to greater verbal memory impairment.^4^ In addition, it is not known whether PDD and DLB differ cognitively in the predementia (ie, mild cognitive impairment [MCI]) stage, because the cognitive profile for the MCI of DLB has yet to be firmly established.^5^ One study found more severe cognitive deficits for patients in the MCI stage of DLB when compared with PD‐MCI.^6^ These data raise questions about whether the early cognitive impairments of DLB and PDD are truly indistinguishable.

*Similar nonmotor profile, with olfactory loss, depression, autonomic dysfunction, and sleep disorders in both*.

Although these nonmotor features are indeed common to PD, PDD, and DLB, their frequency, phenomenology, prominence in the clinical picture, and timing and evolution may differ between PD, PDD, and DLB. Lumping nonmotor symptoms as part of the PD umbrella may deter further investigation into why these differences may emerge and what unique role they may play for DLB and other synucleinopathies.

*Similar imaging, with overlapping patterns of cortical atrophy, glucose utilization, neurotransmitter changes, and diffusion tensor imaging*.

As the authors acknowledged, amyloid deposition imaged with positron emission tomography is higher in DLB when compared with PD and PDD, and other group‐level differences in neuroimaging parameters exist, including more gray matter atrophy on structural MRI in DLB when compared with PDD.^7^, ^8^ These findings therefore underscore some key differences between DLB and PDD on imaging.

*Similar prodromal stage: For example, patients with idiopathic RBD develop both syndromes, with little difference in clinical evolution patterns*.

Rapid eye movement sleep behavior disorder (RBD) clearly can be an early manifestation of Lewy body disease, which eventually evolves into PD or DLB, and many patients with PD and DLB have features of RBD occurring years, or even decades, prior to the onset of parkinsonism cognitive or psychiatric features.^9^ The presence of RBD improves the diagnostic validity of the current DLB criteria.^10^ However, there is insufficient data to substantiate the claim that there is little difference in patterns of clinical evolution between DLB and PDD in those with a prodromal history of RBD.

*Similar genetics: For example, family members with alpha‐synuclein duplication or triplications as well as glucocerebrosidase (GBA) mutations can present with either condition*.

Some of the most compelling evidence to maintain a distinction between PDD and DLB comes from recent studies that have uncovered some genetic differences between them. Specifically, single nucleotide polymorphisms in the gene encoding mitochondrial transcription factor A were found to be associated with PDD and not DLB.^11^ Also, the apolipoprotein e E4 allele frequency was higher in pure DLB (ie, without concomitant AD pathology) than in PDD.^12^ Furthermore, although GBA mutations are reported in both DLB and PD, pure DLB has an intermediate mutation frequency between AD and PD.^13^ A recent genomewide study of DLB also demonstrated different genetic risk factors when compared with PD.^14^

## Addressing the “Defense of the Status Quo” Comments

*Although patients with DLB and PDD look similar at end stage, the course can be extremely different*.

The most agreed on and empirically demonstrated clinical difference between DLB and PDD pertains to clinical course in the context of the temporal onset of dementia and motor signs (ie, the 1‐year rule). This has special relevance when considering the prodromal and preclinical stages of these neurodegenerative conditions. In these conditions, with both motor and cognitive deficits, when parkinsonian motor signs are the initial clinical feature (PDD), the onset of dementia develops an average of 6 years later.^15^, ^16^ When dementia is a presenting feature (as in DLB), the onset of parkinsonism develops an average of 2 years later.^10^ Clinical and pathologic differences are associated with a longer duration of parkinsonism relative to dementia onset and include older age,^17^ later onset of visual hallucinations,^18^ less severe cortical alpha‐synuclein pathology, lower plaque scores, and greater cholinergic cortical deficits^19^ in PDD when compared with DLB. Also, there is a difference in severity as well as phenomenology of motor signs and response to dopaminergic therapy between the typical patient with PDD who has had many years of motor PD when compared with the archetypal patient who fits DLB criteria, whose duration of motor dysfunction is less, severity is often milder, and response to dopaminergic therapy is often less robust.

### Many Parkinsonian DLB Patients Would Not Meet Criteria for Clinical PD

We agree with this statement and would add that in DLB there is also a subset of patients who do not have parkinsonism. These patients meet clinical criteria for probable DLB and have pathologic confirmation of Lewy body disease, but do not exhibit clear‐cut extrapyramidal signs during life.^10^ This is one example of the phenotypic heterogeneity in DLB and an important reason why the most common misdiagnosis of DLB continues to be AD.^20^ More work is needed to better understand DLB without parkinsonism, and placing DLB under the umbrella of PD may deter further investigation of the nonmotor and motor features of DLB.

## Addressing the “Moving Forward” Comments

In the section titled “Moving Forward,” the authors suggest that we consider omitting the 1‐year rule and propose using “PD (DLB subtype)” for those patients who meet clinical criteria for DLB and then fulfill criteria for PD while maintaining the diagnosis of DLB for those who never fulfill criteria for PD. We believe that there is insufficient evidence to support this change currently, and adding an additional diagnostic category will only confuse the distinction further.

We agree that the 1‐year time period is arbitrary, may not be optimal, and will almost certainly be modified in the future when the genetic underpinnings, pathophysiologic mechanisms, and prodromal states of these disorders are better understood. It is well known that identifying a precise date of onset for either parkinsonism or dementia is often impossible, so that the difficulties posed by the hypothetical clinical scenario proposed by the authors, with onset of dementia at 10 months, is likely rare in routine clinical practice and does not justify changing diagnostic criteria at this time. Empirical data are needed to determine whether the use of the 1‐year cut‐off has benefit, or if a longer cut‐off between the onset of parkinsonism and the development of dementia would be a better demarcation of PDD from DLB. Although the 1‐year rule may not be optimal, we argue that modifying the diagnosis of a DLB patient by whether they meet clinical criteria for PD would neither help patients and caregivers understand the prognosis nor serve to inform clinical practice, which was part of the original rationale for this guideline.^21^, ^22^ To reliably distinguish parkinsonism from PD may be achievable in a specialist movement disorder setting, but would likely introduce significant variation across the wide range of different clinical settings in which dementia patients are assessed and diagnosed.

## Implications for Diagnosis, Evaluation, Medical Management, and Social and Supportive Care

The diagnosis, evaluation, medical management, education, and community resources for patients with dementia and their families may be difficult to cover comprehensively when viewed from the perspective that PD (and thereby, by the task force's implication, DLB) is predominantly a movement disorder. Clinical evaluation and the management of people with dementia raise complex issues and have to be delivered by a diversity of clinicians with very different levels of expertise.

Patients, families, and clinicians therefore require a diagnostic system that is simple, has high face validity, and is widely used. Two decades of concerted effort by the international research community has crafted a mutually agreed on approach to the diagnosis of PD, PDD, PD‐MCI, and DLB, and modification of this approach should be clearly supported by empirical data. It is precisely this collaborative stability that has enabled both PDD and DLB to have entered *DSM‐5* 23 and to have been assigned as a national research priority by the United States.^24^

## Summary and Recommendations

We argue that the statements from the 2005 Consensus Group on DLB continue to hold true.^22^ Therefore, we propose the following:

- The 1‐year rule distinguishing PDD from DLB is worth maintaining because it serves an important purpose in clinical practice and clinical and basic science research and in helping the lay community understand the complexity of these apparently different clinical phenotypes. Modification of the 1‐year time period may be justified when the genetic underpinnings, pathophysiologic mechanisms, and prodromal states of these disorders are better understood. Modifying the diagnosis of patients who meet diagnostic criteria for DLB based on whether they meet diagnostic criteria for PD is unjustified at this time, and the addition of a further diagnostic category, “PD (DLB subtype),” is not necessary and is potentially confusing.
- Continued attention should be given to identifying the mild or early manifestations of Lewy body disease and characterizing the longitudinal clinical and biomarker changes associated with evolving Lewy body disease.
- Symptomatic and disease‐modifying interventions should be developed using pharmacologic and nonpharmacologic modalities for the syndromes associated with Lewy body disease.
- Both phenotypes should be included in clinical trials of new therapies for Lewy body dementia (as opposed to lumping them into 1 phenotype) until more research improves knowledge about the differences and similarities between PDD and DLB.

## Authors' Roles

1. Research project: A. Conception, B. Organization, C. Execution; 2. Statistical Analysis: A. Design, B. Execution, C. Review and Critique; 3. Manuscript: A. Writing of the first draft, B. Review and Critique.

B.F.B.: 1A, 3A, 3B

D.W.D.: 1A, 3B

J.E.D.: 1A, 3B

T.J.F.: 1A, 3B

D.R.G.: 1A, 3B

J.E.G.: 1A, 3B

J.G.G.: 1A, 3B

J.H.G.: 1A, 3B

H.I.H.: 1A, 3B

D.I.K.: 1A, 3B

K.K.: 1A, 3B

J.B.L.: 1A, 3B

C.F.L.: 1A, 3B

O.L.L.: 1A, 3B

I.G.M.: 1A, 3B

A.B.S.: 1A, 3B

A.T.: 1A, 3B

D.T.: 1A, 3B

D.W.: 1A, 3B

C.P.Z.: 1A, 3B

## Full financial disclosures of all authors for the past 12 months

B.F.B: serves as an investigator for clinical trials sponsored by GE Healthcare, FORUM Pharmaceuticals and C2N Diagnostics. He receives royalties from the publication of a book entitled Behavioral Neurology Of Dementia (Cambridge Medicine, 2009). He serves on the Scientific Advisory Board of the Tau Consortium. He receives research support from the NIH and the Mangurian Foundation. D.W.D: receives research support from the NIH and the Mangurian Foundation. J.E.D.: receives research support from NIH, the Department of Veterans Affairs and the Michael J. Fox Foundation. T.J.F.: receives research support from the NIH and the Mangurian Foundation. D.R.G.: receives research support from the NIH. J.E.G.: serves as an investigator in clinical trials sponsored by the National Institutes of Health, Axovant, and Biogen. He serves on the DSMB of clinical trials sponsored by NIH and the Alzheimer Drug Discovery Fund. He receives licensing fees from Novartis, Pfizer, and Eisai for co‐creation of the AD8. He receives research support from the NIH, Association for Frontotemporal Degeneration, Florida Department of Health, and the Mangurian Foundation.

J.G.G.: receives research support from the Acadia, NIH, Michael J. Fox Foundation, and Rush University; serves as an investigator for clinical trials sponsored by Biotie and Teva; has been a consultant for Acadia, Pfizer, and Teva; and has received honoraria from the American Academy of Neurology, MedEdicus, Movement Disorders Society, and Pri‐Med.

J.H.G.: receives research support from the NIH and serves on the Scientific Advisory Board of Neurimmune Holding Company. H.I.H.: receives research support from NIH. D.I.K.: receives research support from NIH, AHRQ, Axovant, Guardian Angel Thrift Fund, Janssen Research and Development, Maxwell Family Foundation, Navidea Biopharmaceuticals, Neurim Pharmaceuticals, TauRx; consulting fees from Janssen Research and Development and Takeda. K.K.: serves on the data safety monitoring board for Takeda Global Research & Development Center, Inc.; receives research support from the NIH and Minnesota Partnership for Biotechnology and Medical Genomics. J.B.L.: serves as an investigator for clinical trials sponsored by the Alzheimer's Drug Discovery Foundation and Lundbeck. He is a consultant for Axovant, GE Healthcare, Navidea Biopharmaceuticals, Piramal Healthcare, and Teva. He receives research support from the Alzheimer's Association, Genzyme/Sanofi, Jane and Lee Seidman Fund, Michael J Fox Foundation, and the NIH. C.F.L.: serves as an investigator in clinical trials sponsored by the National Institutes of Health, Avanir, Eli Lilly, Axovant, and Roche. O.L.L.: receives research support from NIH. I.G.M.: receives research support from NIHR and MRC. He provides consultancy to Axovant, GE Healthcare and Takeda. A.B.S.: receives research support from NIH. A.T.: is an employee of the Lewy Body Dementia Association. D.T.: receives research support from NIH and the Department of Veterans Affairs. D.W.: receives research funding or support from Michael J. Fox Foundation for Parkinson's Research, National Institutes of Health (NINDS), Novartis Pharmaceuticals, Department of Veterans Affairs, Avid Radiopharmaceuticals, Alzheimer's Disease Cooperative Study, and the International Parkinson and Movement Disorder Society; honoraria from AbbVie, Acadia, Biogen, Biotie, Clintrex LLC, Janssen, Merck, Novartis, Pfizer, Teva Pharmaceuticals, UCB, and the CHDI Foundation; license fee payments from the University of Pennsylvania for the QUIP and QUIP‐RS; royalties from Wolters Kluweland; and fees for legal consultation for lawsuit related to antipsychotic prescribing in a patient with Parkinson's disease. C.P.Z.: receives research support from the American Parkinson Disease Association, Department of Veterans Affairs, and NIH.

Association, Department of Veterans Affairs, and NIH.

## References

[1] Berg D , Postuma R , Bloem B , et al. Time to redefine PD? Introductory statement of the MDS Task Force on the definition of Parkinson's disease. Mov Disord 2014;29:454–462. 24619848 10.1002/mds.25844 PMC4204150

[2] Mosimann UP , Mather G , Wesnes KA , O'Brien JT , Burn DJ , McKeith IG . Visual perception in Parkinson disease dementia and dementia with Lewy bodies. Neurology 2004;63:2091–2096. 15596755 10.1212/01.wnl.0000145764.70698.4e

[3] Ballard CG , Aarsland D , McKeith I , et al. Fluctuations in attention: PD dementia vs DLB with parkinsonism. Neurology 2002;59:1714–1720. 12473758 10.1212/01.wnl.0000036908.39696.fd

[4] Johnson DK , Morris JC , Galvin JE . Verbal and visuospatial deficits in dementia with Lewy bodies. Neurology 2005;65:1232–1238. 16247050 10.1212/01.wnl.0000180964.60708.c2

[5] Ferman T , Smith G , Kantarci K , et al. Non‐amnestic mild cognitive impairment progresses to dementia with Lewy bodies. Neurology 2013;81:2032–2038. 24212390 10.1212/01.wnl.0000436942.55281.47 PMC3854825

[6] Yoon J , Lee J , Yong S , Moon S , Lee P . The mild cognitive impairment stage of dementia with lewy bodies and Parkinson disease: a comparison of cognitive profiles. Alzheimer Dis Assoc Disord 2014;28:151–155. 24126215 10.1097/WAD.0000000000000007

[7] Edison P , Rowe CC , Rinne JO , et al. Amyloid load in Parkinson's disease dementia and Lewy body dementia measured with [11C]PIB positron emission tomography. J Neurol Neurosurg Psych 2008;79:1331–1338. 10.1136/jnnp.2007.127878 18653550

[8] Gomperts SN . Imaging the role of amyloid in PD dementia and dementia with Lewy bodies. Cur Neurol Neurosci Rep 2014;14:472. 10.1007/s11910-014-0472-6 25011528

[9] Boeve B , Silber M , Ferman T , et al. Clinicopathologic correlations in 172 cases of rapid eye movement sleep behavior disorder with or without a coexisting neurologic disorder. Sleep Med 2013;14:754–762. 23474058 10.1016/j.sleep.2012.10.015 PMC3745815

[10] Ferman T , Boeve B , Smith G , et al. Inclusion of RBD improves the diagnostic classification of dementia with Lewy bodies. Neurology 2011;77:875–882. 21849645 10.1212/WNL.0b013e31822c9148 PMC3162640

[11] Gatt A , Jones E , Francis P , Ballard C , Bateman J . Association of a polymorphism in mitochondrial transcription factor A (TFAM) with Parkinson's disease dementia but not dementia with Lewy bodies. Neurosci Let 2013;557(Pt B):177–180. 24184878 10.1016/j.neulet.2013.10.045

[12] Tsuang D , Leverenz J , Lopez O , et al. APOE epsilon4 increases risk for dementia in pure synucleinopathies. JAMA Neurol 2013;70:223–228. 23407718 10.1001/jamaneurol.2013.600 PMC3580799

[13] Tsuang D , Leverenz JB , Lopez OL , et al. GBA mutations increase risk for Lewy body disease with and without Alzheimer disease pathology. Neurology 2012;79:1944–1950. 23035075 10.1212/WNL.0b013e3182735e9a PMC3484986

[14] Bras J , Guerreiro R , Darwent L , et al. Genetic analysis implicates APOE, SNCA and suggests lysosomal dysfunction in the etiology of dementia with Lewy bodies. Hum Molec Gen 2014;23:6139–6146. 24973356 10.1093/hmg/ddu334 PMC4222357

[15] Evans JR , Mason SL , Williams‐Gray CH , et al. The natural history of treated Parkinson's disease in an incident, community based cohort. J Neurol Neurosurg Psychiatry 2011;82:1112–1118. 21593513 10.1136/jnnp.2011.240366

[16] Aarsland D , Kvaloy JT , Andersen K , et al. The effect of age of onset of PD on risk of dementia. J Neurol 2007;254:38–45. 17508138 10.1007/s00415-006-0234-8

[17] Savica R , Grossardt B , Bower J , Boeve B , Ahlskog J , Rocca W . Incidence of dementia with Lewy bodies and parkinsonism with dementia (P03.097). JAMA Neurol 2013;70:1396–1402. 24042491 10.1001/jamaneurol.2013.3579 PMC4181848

[18] Williams DR , Lees AJ . Visual hallucinations in the diagnosis of idiopathic Parkinson's disease: a retrospective autopsy study. Lancet Neurol 2005;4:605–610. 16168928 10.1016/S1474-4422(05)70146-0

[19] Ballard C , Ziabreva I , Perry R , et al. Differences in neuropathologic characteristics across the Lewy body dementia spectrum. Neurology 2006;67:1931–1934. 17159096 10.1212/01.wnl.0000249130.63615.cc

[20] Nelson P , Jicha G , Kryscio R , et al. Low sensitivity in clinical diagnoses of dementia with Lewy bodies. J Neurol 2010;257:359–366. 19795154 10.1007/s00415-009-5324-y PMC2839040

[21] McKeith I , Galasko D , Kosaka K , et al. Consensus guidelines for the clinical and pathologic diagnosis of dementia with Lewy bodies (DLB): report of the Consortium on DLB International Workshop. Neurology 1996;47:1113–1124. 8909416 10.1212/wnl.47.5.1113

[22] McKeith I , Dickson D , Lowe J , et al. Dementia with Lewy bodies: diagnosis and management: third report of the DLB Consortium. Neurology 2005;65:1863–1872. 16237129 10.1212/01.wnl.0000187889.17253.b1

[23] American Psychiatric Association . Diagnostic and Statistical Manual of Mental Disorders, 5th ed *(DSM‐5)* Arlington, VA: American Psychiatric Publishing; 2013.

[24] Montine T , Koroshetz W , Babcock D , et al. Recommendations of the Alzheimer's disease‐related dementias conference. Neurology 2014;83:851–860. 25080517 10.1212/WNL.0000000000000733 PMC4155046

